# Identification of Circular RNAs Related to Vascular Endothelial Proliferation, Migration, and Angiogenesis After Spinal Cord Injury Using Microarray Analysis in Female Mice

**DOI:** 10.3389/fneur.2021.666750

**Published:** 2021-09-03

**Authors:** Xin Ye, Yilei Chen, Jiasheng Wang, Jian Chen, Ying Yao, Lin-lin Wang, Fengdong Zhao

**Affiliations:** ^1^Department of Neurosurgery, Sir Run Run Shaw Hospital of Zhejiang University School of Medicine, Hangzhou, China; ^2^Department of Orthopaedics, Sir Run Run Shaw Hospital of Zhejiang University School of Medicine, Hangzhou, China; ^3^Department of Neurointensive Care Unit, Second Affiliated Hospital of Zhejiang University School of Medicine, Hangzhou, China; ^4^Department of Basic Medicine Sciences, Department of Orthopaedics of Sir Run Run Shaw Hospital, Zhejiang University School of Medicine, Hangzhou, China

**Keywords:** angiogenesis, circular RNAs, migration, proliferation, spinal cord injury

## Abstract

**Background:** Traumatic spinal cord injury (SCI) can result in severe disability and causes a considerable socio-economic burden worldwide. Circular RNAs (circRNAs) are important regulators of gene expression and pathological processes, and may represent therapeutic targets for SCI. To further evaluate the role of circRNAs in SCI, we elucidated circRNA expression profiles related to vascular endothelial proliferation, migration, and angiogenesis during the early stages of secondary injury in a mouse model of SCI.

**Methods:** Microarray analysis was performed to investigate the circRNA expression patterns in the spinal cord 3 days after SCI in female mice. Bioinformatic analyses, including GO enrichment analysis, KEGG pathway analysis, and circRNA-miRNA-mRNA network construction, were conducted to explore the role of circRNA dysregulation in vascular endothelial proliferation, migration, and angiogenesis following SCI.

**Results:** The expression of 1,288 circRNAs was altered (>2-fold change, *p* < 0.05) in the spinal cord after SCI, consisting of 991 upregulated and 297 downregulated circRNAs. We constructed a circRNA-mRNA network to predict whether these circRNAs could act as “miRNA sponges.” We next assessed the association of altered circRNAs with vascular endothelial proliferation, migration, and angiogenesis using GO and KEGG analyses. Using this analysis, we found that a total of 121 circRNAs were correlated with vascular endothelial proliferation, migration, and angiogenesis in the spinal cord after SCI.

**Conclusions:** Our study provides circRNA expression profiles during the early stages of SCI. circRNA.7079, circRNA.7078, and circRNA.6777 were found to play key roles in the vascular endothelial proliferation, migration, and angiogenesis, and may represent therapeutic targets for SCI.

## Introduction

Traumatic spinal cord injury (SCI) affects ~500,000 people worldwide each year, and its high associated morbidity causes considerable societal burden and socioeconomic impact (1). Patients with SCI often experience devastating neurological impairments and require complex long-term care (2, 3). Non-coding RNAs (ncRNAs) cover more than 98% of the human genome (4), and in the past decade, the biological roles of long non-coding RNAs (lncRNAs) in SCI have been extensively researched, including a large number of studies assessing the regulatory roles of lncRNAs after SCI (5–8). However, the roles of circular RNAs (circRNAs) in SCI are barely known. circRNAs represent a new class of endogenous non-coding RNAs which regulate gene expression in mammals. circRNAs can function as a miRNA sponge to relieve miRNA suppression, thus promoting mRNA translation. For example, CDR1 as sponging miR-7 is related with pathogenesis of lung cancer, breast cancer, glioma, and amyotrophic lateral sclerosis, etc. Contrary to linear RNA, circRNA contains a covalently closed-loop structure without a 5′-3′ polarity and a poly (A) tail (9, 10). Numerous circRNAs are highly conserved, expressed specifically in cell types or developmental stages (11, 12), and have been implicated in the development and pathogenesis of several diseases, including Alzheimer's disease (13).

Recent studies have proposed a significant circRNA dysregulation in the central nervous system following SCI, which may also contribute to disrupted vascular endothelial proliferation, migration, and angiogenesis in SCI (14). circRNAs may therefore represent a new kind of biomarker and therapeutic target for SCI angiogenesis.

In this study, we applied circRNA microarrays to explore circRNA expression levels following SCI in female mice, and subsequently carried out gene ontology (GO) and pathway analyses to further evaluate the function of circRNAs after SCI. Using ceRNA analysis of the circRNA-miRNA-mRNA network, we identified three circRNAs for which related mRNAs may affect the expression of target mRNAs by sponging miRNA. We furthermore predicted miRNAs which could be regulated by these differentially expressed circRNAs using microRNA-circRNA-mRNA network analysis.

## Materials and Methods

### Animals

Young adult C57BL/6 mice (female, 25–30 g, 8 weeks) were housed for at least seven days in a controlled temperature environment (23°C) on a 12 h light/dark cycle before the operation. The mice were allowed *ad libitum* access to water and food except on the day before the operation, on which the mice were allowed only *ad libitum* access to water. All animal procedures were approved by the Institutional Animal Care and Use Committee of the Sir Run Run Shaw Hospital of Zhejiang University School of Medicine, People's Republic of China.

### SCI Model

The SCI model used Allen's method, as described in our previous study (15). Twelve mice were anesthetized intraperitoneally by 1% pentobarbital sodium. They were randomly divided into two groups: SCI group (*n* = 6) and sham group (*n* = 6). The dorsal surface of thoracic spinal cord segments was exposed, taking care to avoid any dural tears at the T9-T10 level after laminectomy. Subsequently, a New York University (NYC) II struck instrument with a 1-mm-diameter impactor from a height of 25 mm was used to induce a moderate contusion injury of the spinal cord at T10. The wound was then closed by suturing the muscle layer using nylon sutures. The same procedure without the contusion injury was performed in the sham group.

### RNA Extraction and Purification

Three days after SCI, six mice (SCI group, *n* = 3; sham group, *n* = 3) were anesthet ized. One cm long segments at the T9-T10 spinal cord enclosing the injured site were rapidly frozen in liquid nitrogen and transported to Shanghai Biotechnology Corporation for microarray analysis on dry ice. Total RNA was extracted and purified using the mirVana™ miRNA Isolation Kit (Cat#AM1561, Ambion, Austin, TX, US) following the manufacturer's instructions and the RIN was assessed on the Agilent Bioanalyzer 2,100 (Agilent technologies, Santa Clara, CA, US).

### Microarray Analysis

Total RNA was amplified and labeled using the Low Input Quick Amp Labeling Kit, One-Color (Cat.#5190-2305, Agilent technologies, Santa Clara, CA, US) following the manufacturer's instructions. Labeled cRNAs were purified using the RNeasy mini kit (Cat. #74106, QIAGEN, GmBH, Germany). Each slide was hybridized with 1.65 μg of Cy3-labeled cRNA using the Gene Expression Hybridization Kit (Cat. #5188-5242, Agilent technologies, Santa Clara, CA, US) in a Hybridization Oven (Cat. #G2545A, Agilent technologies, Santa Clara, CA, US). Slides were then washed in staining dishes according to the manufacturer's instructions (Cat. #121, Thermo Shandon, Waltham, MA, US) with the Gene Expression Wash Buffer Kit (Cat. #5188-5327, Agilent technologies, Santa Clara, CA, US) after 17 h hybridization. Slides were scanned on a Agilent Microarray Scanner (Cat#G2565CA, Agilent technologies, Santa Clara, CA, US) with default settings, as follows: Dye channel: Green, scan resolution = 3μm, PMT 100%, 20 bit. Raw data were normalized using the quantile algorithm of the *limma* package in R. Data were extracted using the Feature Extraction software 12.0 (Agilent technologies, Santa Clara, CA, US).

### Bioinformatics Analysis

Scanned images were imported into the Agilent Feature Extraction software for raw data extraction. Using the R software package *limma*, quantile normalization of raw data and data processing was conducted and low intensity probe filtering was performed. The log2-ratio was used for quantile normalization. Via fold change filtering or volcano plot filtering, differentially expressed circRNAs with statistical significance were identified. Hierarchical clustering was performed to highlight differential circRNA expression patterns among different samples. Significantly differentially expressed circRNAs were selected as follows: Fold change ≥2, *p*-value <0.05. We entered the host genes of differentially expressed circRNAs into the Database for Annotation, Visualization, and Integrated Discovery (DAVID; http://david.abcc.ncifcrf.gov) for annotation and functional analysis. GO analysis including the categories of biological process, cellular component, and molecular function was performed on account of GO (http://www.geneontology.org). The significance of GO term enrichment was indicated by fold change and *P*-value (FC ≥2 and *P* < 0.05 were considered statistically significant). We also used pathway analysis to classify differentially expressed genes on account of the Kyoto Encyclopedia of Genes and Genomes (KEGG) database to predict pathways of interest. mRNAs related to vascular endothelial proliferation, migration, and angiogenesis from the mRNAs which changed more than 2-fold and a *P*-value < 0.05 were chosen. We then assessed circRNA correlation with angiogenesis via cis-acting mRNA networks.

GO analysis, which was performed to explore the potential functions of parental genes in terms of the circRNAs, covered three different aspects: Biological process (BP), cellular component (CC), and molecular function (MF). The top 10 enriched GO terms and pathways among the two groups were ranked by enrichment score [-log10 (*P*-value)] as identified by the Database for Annotation, Visualization, and Integrated Discovery (DAVID; http://www.david.abcc.ncifcrf.gov/).

### qRT-PCR Validation

qRT-PCR was used to validate circRNA expression patterns following microarray detection. Briefly, using the ReverTra Ace qPCR Kit (TOYOBO, FSQ-101) and High Capacity cDNA Reverse Transcription Kit with RNase Inhibitor (ABI, 4374966), we reverse-transcribed total RNA. qRT-PCR was conducted in the 7,900 HT Sequence Detection System (ABI, USA) and Quant Studio 5 Real-Time PCR System (ABI, USA) using Power SYBR Green PCR Master Mix (ABI, 4368708). We used Glyceraldehyde 3-phosphate dehydrogenase (GAPDH) as an internal control to normalize the data. The primers of these circRNAs and GAPDH are presented in Table 1.

**Table 1:** The top 20 differentially expressed CircRNAs after spinal cord injury (SCI).

**circRNA**	**Fold change**	**Regulation**	**Chromosome**	**Strand**	**Host Gene**	***p*** **-value**
circRNA.7079	62.85875487	Up	chr14	+	Lgals3	0.004436
circRNA.7078	31.09694285	up	chr14	+	Lgals3	0.011314
circRNA.6777	21.70369315	up	chr14	+	Pbk	0.032084
circRNA.17120	19.44192413	up	chr1	+	Cd84	0.030409
circRNA.10854	18.12036937	up	chr11	−	Top2a	0.020119
mmu-circRNA483	17.26274244	up	chr19	+	Ms4a6c	0.016317
circRNA.27570	17.1963371	up	chr2	−	Cd44	0.008235
circRNA.27321	16.0267925	up	chr2	+	Knstrn	0.042293
circRNA.538	14.81831851	up	chr19	+	Ms4a6c	0.003396
circRNA.8591	13.55325989	up	chr13	−	n/a	0.000530
circRNA.6335	4.509393859	down	chr15	+	C1qtnf3	0.0019097
mmu_circ_0010846	3.761647531	down	chr3	−	Bcan	0.0048737
circRNA.16626	3.673922594	down	chr1	−	Col19a1	0.0016594
circRNA.12533	3.436555425	down	chr11	−	Tenm2	0.0367338
circRNA.6638	3.15487569	down	chr14	+	n/a	0.0340403
circRNA.27485	3.15149557	down	chr2	−	Ano3	0.0114666
circRNA.21851	3.027841911	down	chr5	+	Ksr2	0.0297605
circRNA.4430	3.010502122	down	chr16	−	Prodh	0.0002868
mmu_circ_0012585	2.961510524	down	chr5	+	Rgs12	0.0131907
circRNA.16625	2.958448619	down	chr1	−	Col19a1	0.0384322

### Construction of circRNA-miRNA-mRNA Networks

circRNA microarray expression profiling and data bioinformatics analysis were performed by Shanghai Biotechnology Corporation, Shanghai, People's Republic of China. Based on the expression signals, we built a circRNA-miRNA-mRNA interaction network using a regression model and seed sequence matching analysis. The circRNA-mRNA interactions were predicted using the principle of high related coefficient and at least one perfect seed-matching sequence.

### Western Blotting Analysis

Three days after SCI, six mice (SCI group, *n* = 3; sham group, *n* = 3) were euthanized via an overdose of 1% pentobarbital sodium and the spinal cord lesion site was extracted and flash-frozen at −80°C. The spinal cords were triturated using Tissue Prep (Gering Scientific Instruments, Beijing, China) and cells were dissolved in WB and IP lysis buffer containing phenylmethanesulfonylfluoride (PMSF) and phosphatase inhibitor and centrifuged at 1.3 × 10^4^ g for 15 min at 4°C. Subsequently, the protein concentration was quantified using a BCA kit (Beyotime Institute of Biotechnology, Jiangsu, China). All samples (20 μl) were separated on a SurePAGE^TM^ (Cat.M00653, GenScript®, USA) and transferred onto nitrocellulose membranes. Membranes were blocked in 5% skimmed milk powder dissolved in TBS-T for 1 h. Mouse anti-CD31 (Cat# 12242S, ABclonal, 1:1000), rabbit anti-VEGF (Cat# 3495S, Cell Signaling Technology, 1:1000), and rabbit anti-VEGFR2 (Cat# 4108S, ABclonal, 1:1000) were used for probing interested proteins at 4°C overnight. Membranes were incubated with infrared-labeled secondary antibodies (Li-COR Biosciences) after several washes with TBS-T. In order to visualize the immunoblot bands, an Odyssey infrared imaging system (LI-COR® Biosciences, NE, USA) was used. Band intensity was analyzed with an Image Studio Ver 5.2 system and standardized to the β-Actin internal standard.

### Statistical Analyses

All quantitative data are presented as mean ± standard deviation (mean ± SD). Student's *t*-test was used to calculate the statistical significance between the groups. All statistical analyses were performed using the SPSS 20.0 software (SPSS, Chicago, IL). The correlation of two variables was assessed using the Pearson product-moment correlation coefficient. Genes changing equal to or more than 2-fold with *P* < 0.05 were considered statistically significant.

## Results

### circRNAs Expression Profiles in the Spinal Cord After SCI

**Figure 1:**
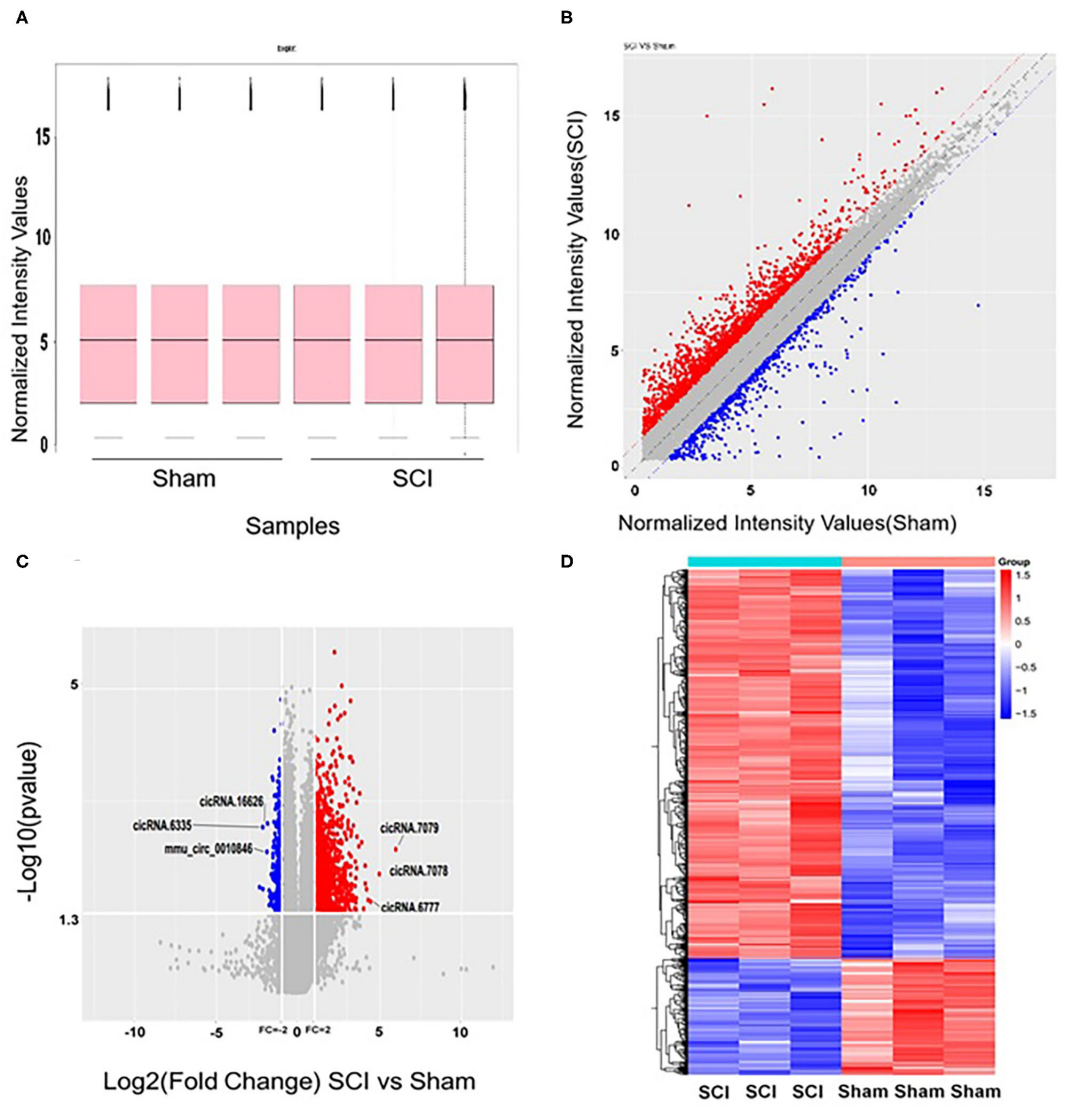
Altered expression profiles of circRNAs in the spinal cord after spinal cord injury (SCI). **(A)** No significant differences were observed in the distributions of the total expression of circRNAs between the two groups. **(B)** The oblique lines are fold change lines. The circRNAs above the top red line and below the bottom blue line indicate a >2-fold change of circRNAs between the two groups. **(C)** Volcano plot shows circRNAs altered in the SCI group compared with sham. **(D)** Differences in circRNA expression. Heat map presenting the circRNA expression profiles; each column represents a sample and each row represents a gene. High relative expression is indicated by red, and low relative expression by blue.

**Figure 2:**
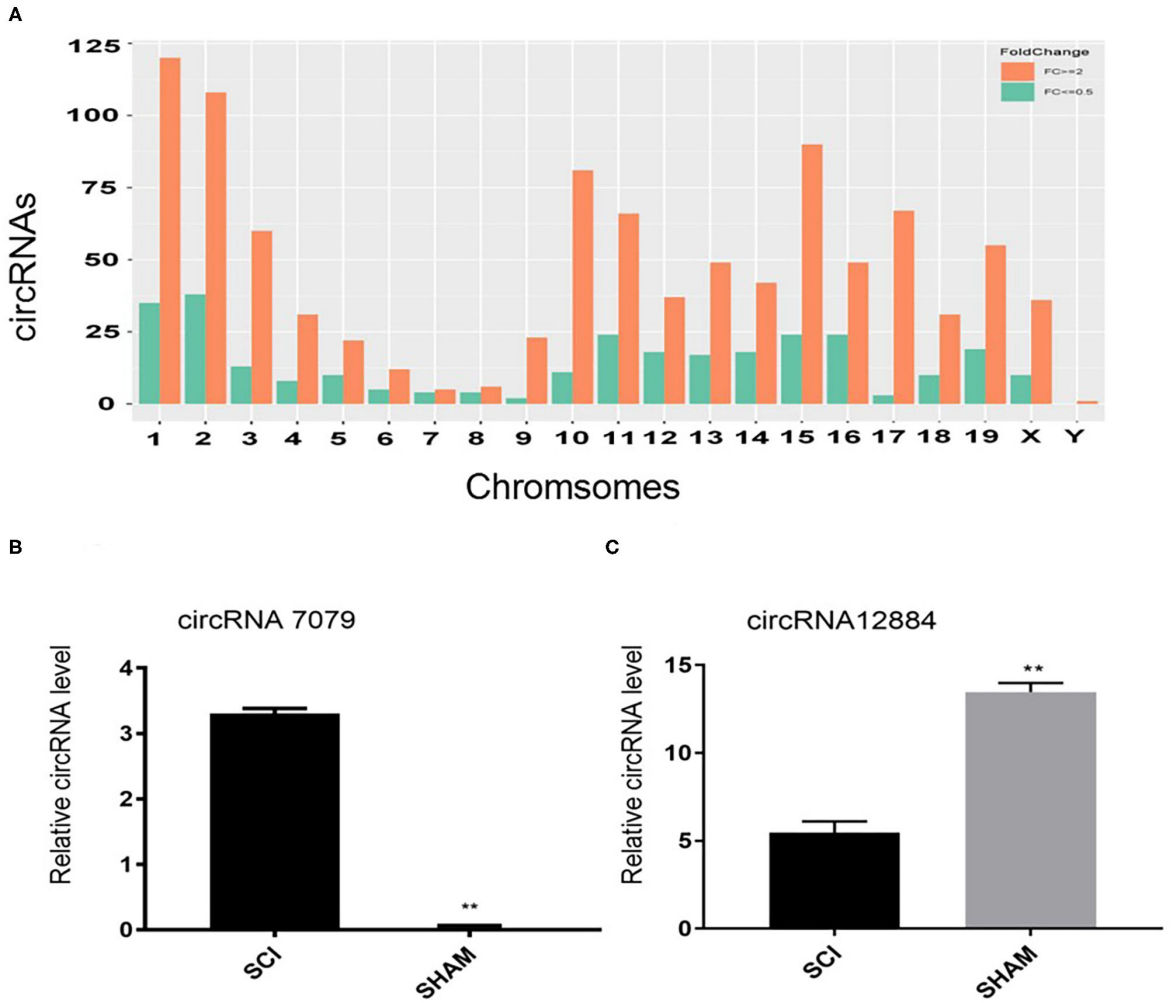
**(A)** The distributions of altered circRNAs on mouse chromosomes. **(B, C)** Real-time polymerase chain reaction (PCR) validation results of two selected circRNAs (circRNA.7079 and circRNA.12884). Data are presented as mean ± SD (*n* = 3 per group). **p < 0.01 significant compared with the SCI group (Student's *t*-test).

The microarrays used in the study contained probes for 37,852 mouse circRNAs, of which 9364 circRNAs were found on circBase, 378 circRNAs were found on Deepbase, and the remaining were found on Shbio. As shown in the box plot in Figure 1A, the distribution of circRNA expression profiles was not different in samples, indicating that post-injury changes observed in the levels of individual circRNAs were not due to chance. Differentially expressed circRNAs were filtered by fold change and *p*-value (>2-fold change, *p* < 0.05), which are illustrated as red and blue dots on the scatter plot in Figure 1B and volcano plot in Figure 1C. Hierarchical clustering was performed to highlight distinguishable circRNA expression patterns between the two groups (Figure 1D). The altered circRNAs were clearly separated into two clusters, indicating that the samples had good intra-group consistency, while circRNA expression levels in the SCI group were significantly different compared to the sham group. Compared with the sham group, 1288 circRNAs were significantly altered (>2-fold change, *p* < 0.05) in the spinal cord after SCI, of which 991 were upregulated and 297 were downregulated. The top 20 altered circRNAs are listed in Table 1. The distributions of altered circRNAs in mice revealed that the upregulated circRNAs were transcribed from all chromosomes, while downregulated circRNAs were transcribed from all chromosomes except chrY (Figure 2A).

### Real-Time PCR Confirmed circRNAs Expression Alteration

In order to verify the microarray data, real-time PCR was used to evaluate the expression of two randomly selected circRNAs. Primers used for qRT-PCR of these circRNAs are listed in Table 2. Compared with the sham group, circRNA.7079 was upregulated in the injured group (Figure 2B), while circRNA.12884 was downregulated significantly after SCI (Figure 2C).

**Table 2:** Primers used for qRT-PCR of CircRNAs after spinal cord injury (SCI).

**Gene**	**Primer name**	**Sequence (5′ to 3′)**
GAPDH (Mouse)	Gapdh-F	TCCTGCACCACCAACTGCTTAG
	Gapdh-R	AGTGGCAGTGATGGCATGGAC
circRNA.7079	CUST_110069_PI435794180-F	CCCGCTTCAATGAGAACAACA
	CUST_110069_PI435794180-R	GGAGCCTGTCCTGGTTATTGTC
circRNA.12884	CUST_118563_PI435794180-F	CTTGTGTCCAAGTGAATGAATGG
	CUST_118563_PI435794180-R	GCCGGTGTCGAGTGAAGAA

### GO Analysis and KEGG Pathway Analysis

**Figure 3:**
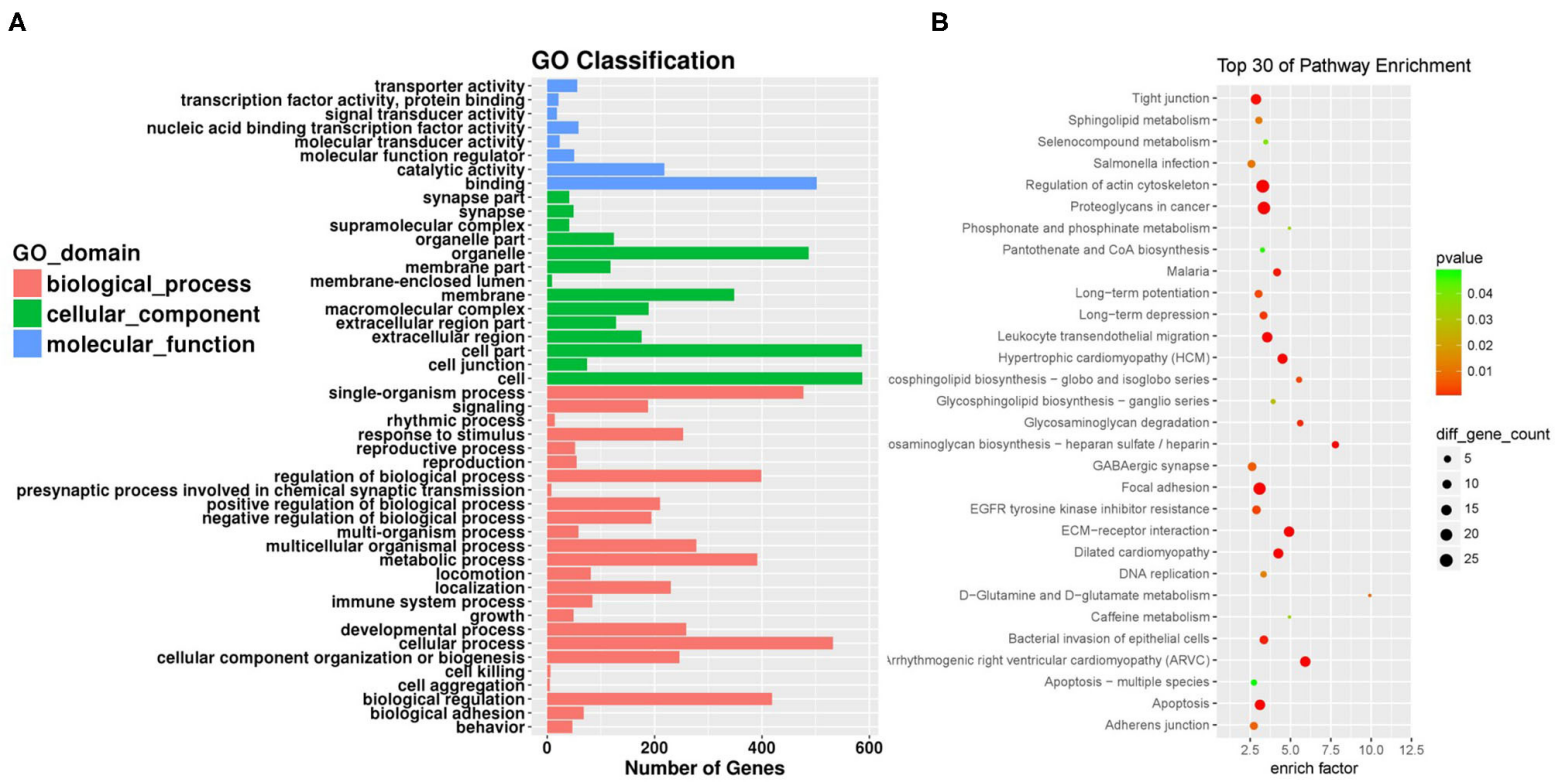
Gene ontology (GO) analysis and Kyoto Encyclopedia of Genes and Genomes (KEGG) pathway annotation of differentially expressed circRNAs. **(A)** The bar plot shows the major enriched and meaningful GO terms of biological process, cellular component, and molecular function related to the altered circRNAs ranged according to the predicted *p*-values. **(B)** The bubble chart shows the top 30 most significantly enriched pathways after SCI. The enrichment factor on the X-axis indicates the specificity of a pathway activating in SCI samples compared with sham samples. The bubble size represents the number of circRNAs enriched in a pathway. Red bubbles indicate a more significant pathway.

In order to analyze the pathways associated with endothelial proliferation, migration, and angiogenesis and assess the numbers of related circRNAs, we performed KEGG analyses. In order to reveal the mRNAs and circRNAs correlated with vascular endothelial proliferation, migration, and angiogenesis, we performed GO analyses. We conducted GO and KEGG pathway analysis for the differentially expressed circRNAs based on their host genes in order to investigate potential biological functions. The GO analysis indicated that “biological regulation,” “cellular process,” and “single-organism process response” were the main related biological processes involved in SCI (Figure 3A). In terms of cellular components, “cell,” “cell part,” and “organelle” were highly activated. Most of the molecular functions activated after SCI were “binding,” “catalytic activity,” and “molecular function regulator.” KEGG analysis showed that the top 30 pathways affected by the altered circRNAs might be involved in the progression of SCI (Figure 3B). The host-genes of circRNAs were predominantly enriched for glycosaminoglycan biosynthesis and extracellular matrix (ECM)-receptor interaction pathways. The most significant pathway was the ECM-receptor interaction, which also had a high enrichment score, reflecting that this pathway was affected in the spinal cord after SCI more than in other diseases or conditions.

### circRNA-miRNA-mRNA ceRNA Network Prediction and Annotation

**Figure 4:**
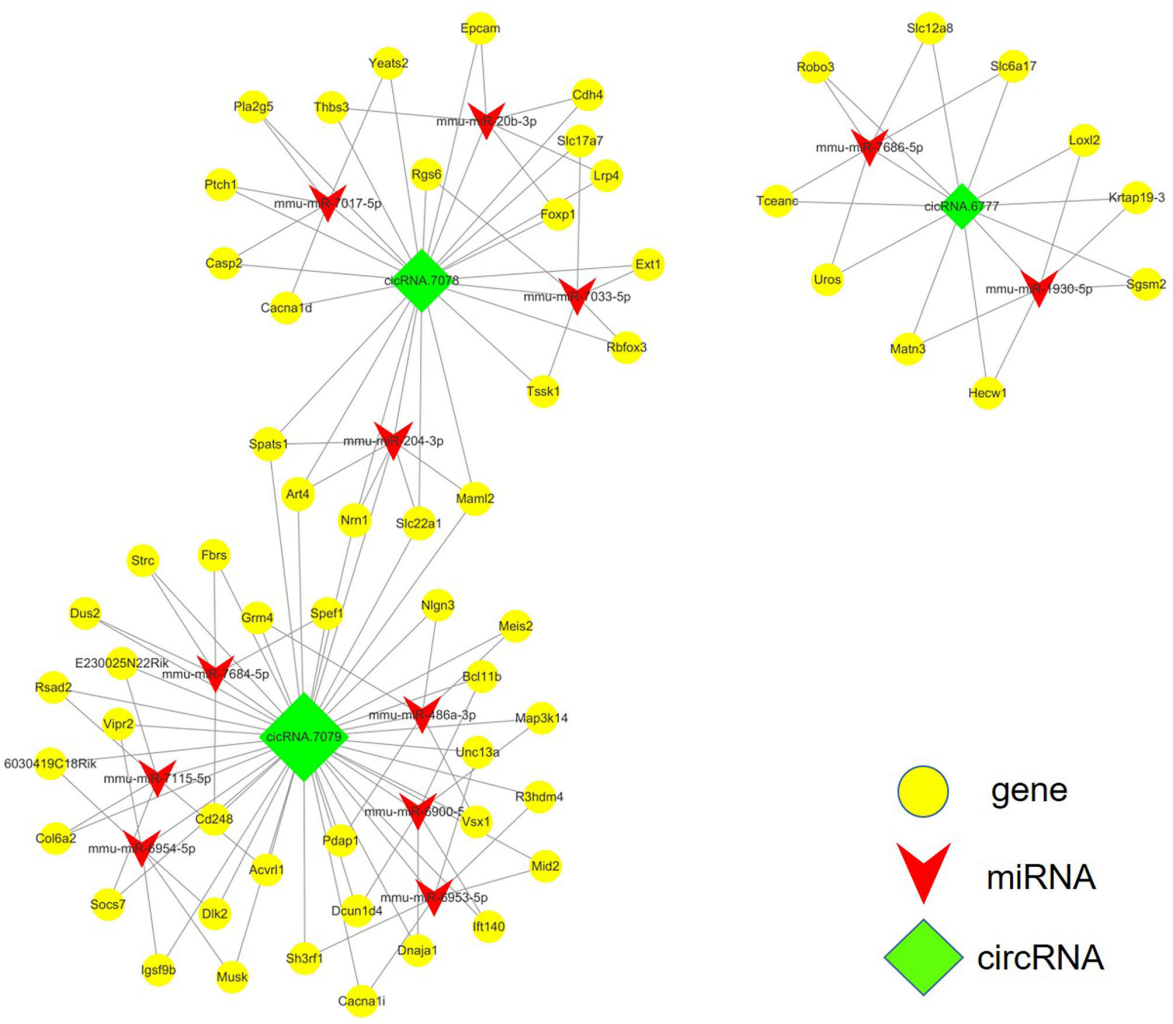
ceRNA analysis of the circRNA-miRNA-mRNA crosstalk network in the mouse spinal cord after SCI. circRNA.7079, circRNA.7078, and circRNA.6777 are plotted as green rhombi. miRNAs targeted by circRNAs are plotted as red arrows in the network, while mRNAs which may be regulated by these three circRNAs are illustrated as yellow dots.

The competing-endogenous RNA (ceRNA) network analysis proposes that RNA transcripts, both coding and non-coding, crosstalk with and coregulate each other using microRNA response elements (MREs). The ceRNA network analysis tremendously expands functional information of coding and non-coding RNAs. Mounting evidence has shown that various types of RNAs, including pseudogenes, long non-coding RNAs, circular RNAs, and messenger RNAs, can function as ceRNAs in distinct physiological and pathophysiological states. We screened the top 20 differently expressed circRNAs after SCI and identified three of them for which related mRNAs could be predicted via ceRNA analysis, circRNA.7079, circRNA.7078, and circRNA.6777. The integrated circRNA-miRNA-mRNA network is shown in Figure 4.

### Vascular Endothelial Proliferation, Migration, and Angiogenesis-Related circRNAs Prediction

**Figure 5:**
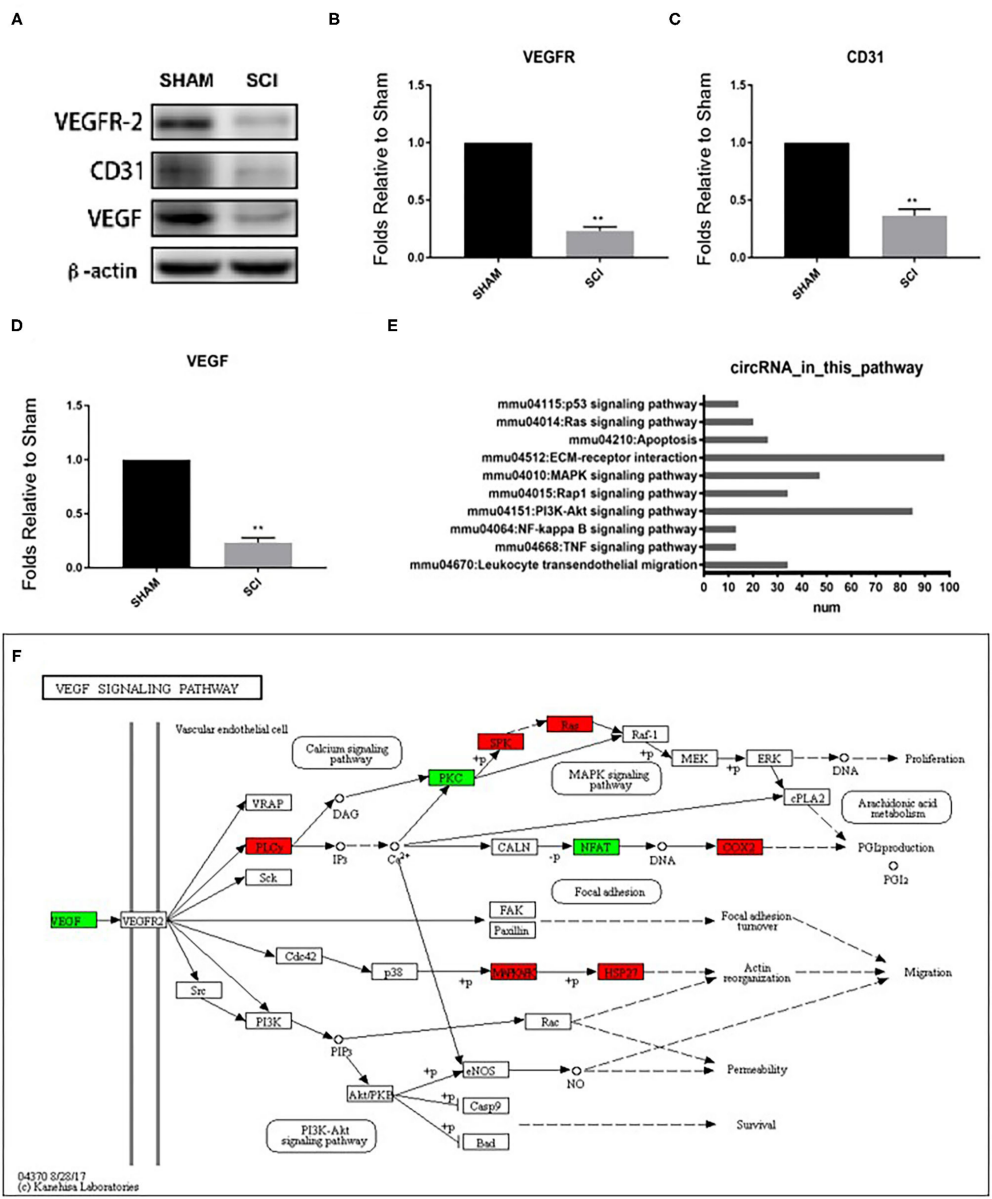
Detection of vascular endothelial proliferation, migration, and angiogenesis responses after SCI and quantification of CD31, VEGF, and VEGFR protein levels 3 days post SCI by western blot **(A–D)**. Analyses of the protein bands measured normalized to the signal of β-actin. Expression levels of proteins in the SCI group are expressed as fold change compared to sham values. **p < 0.01 significant compared with the sham group. Data are presented as the mean ± SEM, *n* = 5. **(E)** The number of circRNAs participating in the pathways associated with vascular endothelial proliferation, migration, and angiogenesis based on KEGG results. **(F)** KEGG map04370 shows the VEGF pathway.

We were interested in the expression profiles of circRNA which correlated with vascular endothelial proliferation, migration, and angiogenesis after SCI, thus datasets concerning other functions were excluded. Firstly, to confirm angiogenesis-related protein expression in the spinal cord, western blotting was performed to detect the expression of angiogenesis-related proteins CD31, VEGF, and VEGFR2 3 days after SCI (Figure 5A). The expression levels of CD31, VEGF, and VEGFR2 were strongly decreased at 3 days after SCI (0.390 ± 0.055-fold of sham, 0.255 ± 0.044-fold of sham, and 0.261 ± 0.034-fold of sham, *P* < 0.001 for all; Figures 5B–D), revealing a reduction of angiogenesis. Next, via KEGG analysis, we analyzed the pathways associated with endothelial proliferation, migration, and angiogenesis and assessed the numbers of related circRNAs (Figure 5E). The host-genes of circRNAs were predominantly enriched for ECM-receptor interaction (*n* = 97) and the PI3K-Akt signaling pathway (*n* = 85), as shown in Figure 5E. In the VEGF signaling pathway, several host-genes of circRNAs, such as PLCy, SPK, Ras, COX2, MAPKAFK, and HSP27 were upregulated, while PKC and NEAT were downregulated (KEGG mmu04370, Figure 5F). VEGFR2 is the main receptor of VEGFA which then triggers vascular endothelial proliferation, migration, and angiogenesis through the VEGF signaling pathway, as shown in KEGG mmu04370.

**Figure 6:**
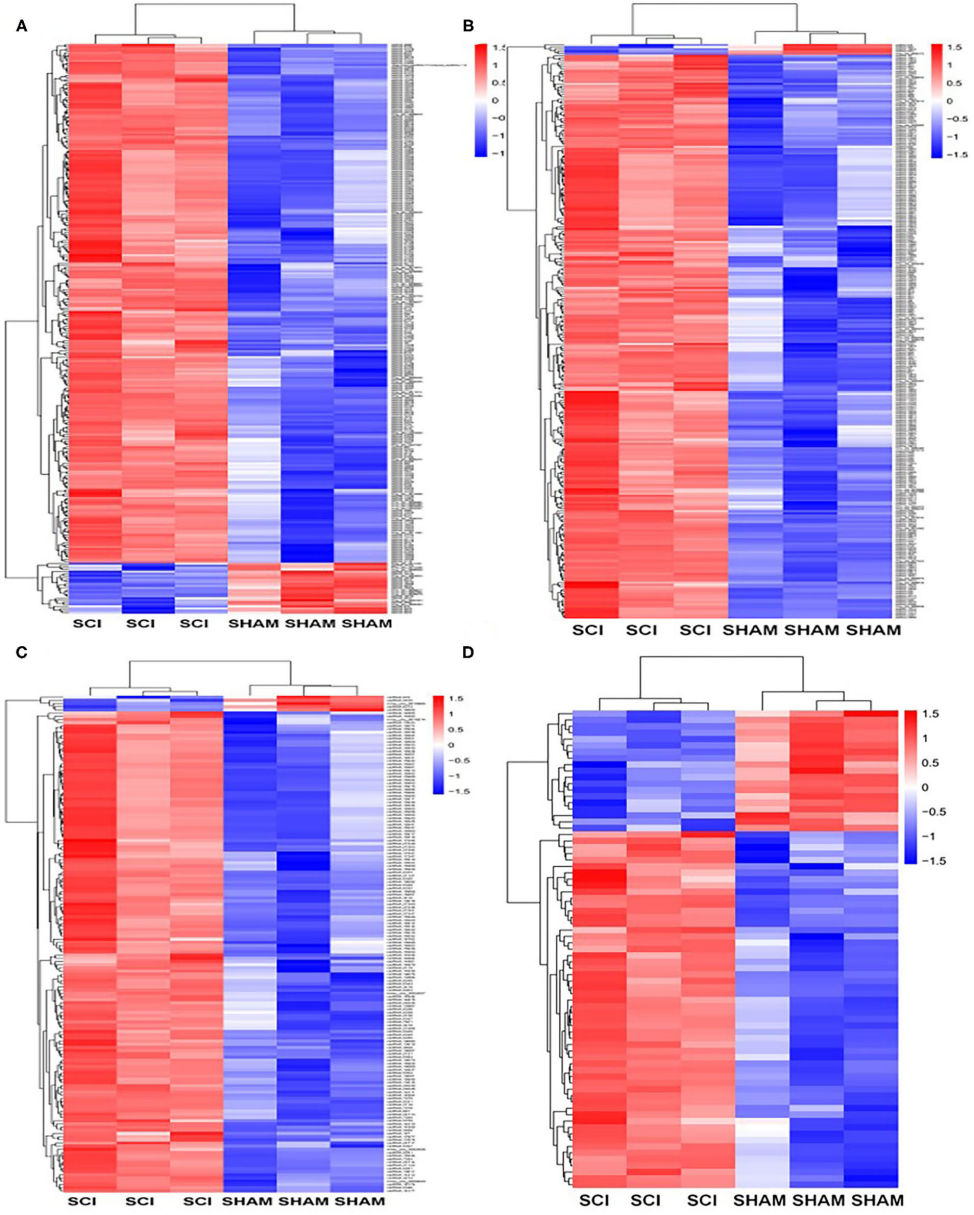
GO analysis for genes and circRNAs related to vascular endothelial proliferation, migration, and angiogenesis. **(A)** The heat map of GO analysis mRNAs (>2-fold change, *p* < 0.01) enriched for the biological processes, cellular components, and molecular function associated with vascular endothelial proliferation. **(B)** Heat map of GO analysis mRNAs (>2-fold change, *p* < 0.01) enriched for biological processes, cellular components, and molecular function associated with vascular endothelial migration and angiogenesis. **(C)** The heat map of GO analysis mRNAs (>2-fold change, *p* < 0.01) enriched for the biological processes, cellular components, and molecular function associated with vascular endothelial angiogenesis. **(D)** The heat map of associated circRNAs (>2-fold change, *p* < 0.01) based on GO analysis of mRNAs.

**Figure 7:**
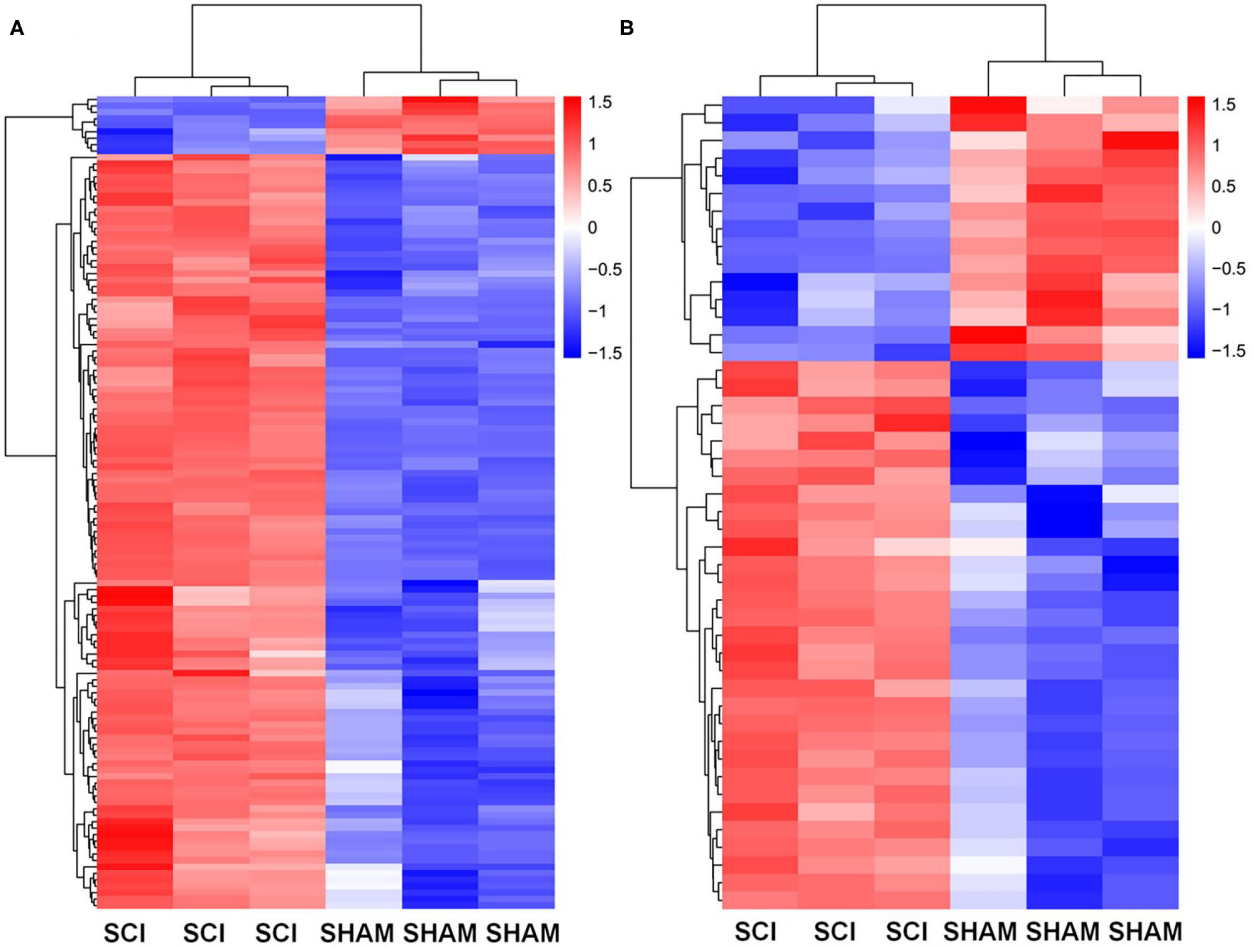
KEGG analysis for genes and circRNAs related to vascular endothelial proliferation, migration, and angiogenesis express profiles. **(A)** Heat map of KEGG analysis mRNAs (>2-fold change, *p* < 0.01) enriched for pathways associated with vascular endothelial proliferation, migration, and angiogenesis. **(B)** The heat map of circRNAs (>2-fold change, *p* < 0.01) based on ceRNA analysis of KEGG mRNA analysis.

Next, the mRNAs and circRNAs related to vascular endothelial proliferation, migration, and angiogenesis were analyzed using GO analyses. This revealed that 191 mRNAs (exhibiting a >2-fold change and *p* < 0.01) were correlated with vascular endothelial proliferation, migration, and angiogenesis; such as “vascular endothelial growth factor production (GO:0010573),” “regulation of vascular endothelial growth factor production (GO:0010574),” “positive regulation of angiogenesis (GO:0045766),” “regulation of angiogenesis (GO:0045765),” and “angiogenesis (GO:0001525),” “sprouting angiogenesis (GO:0002040),” amongst other terms (Figures 6A–C). Seventy-five circRNAs (>2-fold change, *p* < 0.01) were speculated to be correlated with these three biological processes using ceRNA analysis (Figure 6D). In addition, we found 127 mRNAs (>2-fold change, *p* < 0.01) correlated with vascular endothelial proliferation, migration, and angiogenesis based on KEGG analyses, such as “ECM-receptor interaction (PATHWAYID: mmu04512),” “Ras signaling pathway (PATHWAYID: mmu04014),” “PI3K-Akt signaling pathway (PATHWAYID: mmu04151),” and “VEGF signaling pathway (PATHWAYID: mmu04370),” amongst other terms (Figure 7A). Forty-six circRNAs (>2-fold change, *p* < 0.01) were predicted to correlate with these mRNAs using ceRNA analysis (Figure 7B). The top 10 upregulated circRNAs were circRNA.7079, circRNA.7078, circRNA.27570, circRNA.26810, circRNA.5566, circRNA.27348, circRNA.6727, circRNA.26807, circRNA.15929, and circRNA.27347. Among these top 10 circRNAs, circRNA.7079, circRNA.7078, circRNA. 27348, and circRNA.15929 were predicted to be involved in vascular endothelial proliferation, migration, and angiogenesis. circRNA.27570 and circRNA.26810 were predicted to be correlated with both vascular endothelial proliferation and migration, while circRNA.5566 and circRNA.6727 were predicted to be involved in vascular endothelial proliferation only. Thus, these circRNAs were predominantly involved in angiogenesis signaling pathways, such as the PI3K-Akt signaling pathway, the STAT3 signaling pathway, the TCR signaling pathway, and the Wnt/β-catenin signaling pathway (Table 3).

**Table 3:** The top 10 differentially expressed CircRNAs involved in vascular endothelial proliferation, migration, and angiogenesis after SCI.

**circRNA**	**Fold change**	**Regulation**	**miRNA**	**Target gene**	**Downstream pathways or biological processes**	**Biological functions**
circRNA.7079	62.85875487	Up	mmu-miR-761	Lgals3	Wnt/β-catenin signaling pathway	Proliferation/Migration/Angiogenesis
			mmu-miR-1907	Lgals3	Wnt/β-catenin signaling pathway	Proliferation/Migration/Angiogenesis
			mmu-miR-326-5p	BIRC5	PI3K-Akt signaling pathway	Proliferation/Migration/Angiogenesis
			mmu-miR-3474	Pbk	Pbk/Akt signaling pathway	Proliferation/Migration/Angiogenesis
circRNA.7078	31.09694285	Up	mmu-miR-761	Top2a	Her2 signaling pathway	Proliferation/Migration/Angiogenesis
			mmu-miR-1907	Ms4a6c	TCR signaling pathway	Proliferation
			mmu-miR-3474	Cd44	Wnt/β-catenin signaling pathway	Proliferation/Migration/Angiogenesis
			mmu-miR-5710	Knstrn	TCR signaling pathway	Proliferation
circRNA.27570	17.1963371	Up	mmu-miR-9768-3p	Cd44	Wnt/β-catenin signaling pathway	Proliferation/Migration/Angiogenesis
circRNA.26810	13.10873913	Up	mmu-miR-709	BIRC5	PI3K-Akt signaling pathway	Proliferation/Migration/Angiogenesis
			mmu-miR-1956	Lgals3	Wnt/β-catenin signaling pathway	Proliferation/Migration/Angiogenesis
			mmu-miR-1935	Vim	STAT3 signaling pathway	Proliferation/Migration/Angiogenesis
circRNA.5566	12.04671542	Up	mmu-miR-721	Pten	PI3K-Akt signaling pathway	Proliferation/Migration/Angiogenesis
			mmu-miR-1953	Pten	PI3K-Akt signaling pathway	Proliferation/Migration/Angiogenesis
circRNA.27348	11.91692033	Up	mmu-miR-695	Thbs1	STAT3 signaling pathway	Proliferation/Migration/Angiogenesis
			mmu-miR-763	Pten	PI3K-Akt signaling pathway	Proliferation/Migration/Angiogenesis
circRNA.6727	11.8892439	Up	mmu-miR-871-3p	Tnfrsf10b	Akt Signaling Pathway.	Proliferation/Migration/Angiogenesis
			mmu-miR-1190	Pten	PI3K-Akt signaling pathway	Proliferation/Migration/Angiogenesis
circRNA.26807	11.54191573	Up	mmu-miR-709	BIRC5	PI3K-Akt signaling pathway	Proliferation/Migration/Angiogenesis
			mmu-miR-1935	Pten	PI3K-Akt signaling pathway	Proliferation/Migration/Angiogenesis
circRNA.15929	11.52291449	Up	mmu-miR-450b-3p	Fcer1g	leukocyte migration; cytokine production	Proliferation/Migration/Angiogenesis
			mmu-miR-1843b-5p	Fn1	TGF-β signaling pathway	Proliferation/Migration/Angiogenesis
circRNA.27347	11.31077366	Up	mmu-miR-125a-5p	Irf5	Wnt signaling pathway	Proliferation/Angiogenesis
			mmu-miR-343	Thbs1	Wnt/β-catenin signaling pathway	Proliferation/Migration/Angiogenesis
			mmu-miR-691	Pten	PI3K-Akt signaling pathway	Proliferation/Migration/Angiogenesis

## Discussion

Recently, the use of stem cell transplantation, olfactory ensheathing cell transplantation, and tissue engineering technology to promote the repair of SCI has brought hope to this field, and the endogenous molecular mechanism in traumatic injury has been gradually revealed. Studies have described that altered levels of specific circRNAs are involved in brain injury, ischemia, and stroke (16–19). In this study, we have elucidated the expression profiles of circRNAs in a mouse model of SCI.

We assessed the top 20 differentially expressed circRNAs between sham and SCI, and identified 3 circRNAs for which related mRNAs could be predicted via ceRNA analysis: circRNA.7079, circRNA.7078, and circRNA.6777. The circRNA with the top fold change was circRNA.7079, and its expression alteration was also confirmed by qRT-PCR. It was interesting to note that circRNA.7079 might be involved in the regulation of the mRNA of BIRC5, Lgals3, and Pb; this will need to be validated in the future. Moreover, ceRNA analysis of the circRNA-miRNA-mRNA network predicted that circRNA.7079 may affect the expression of target mRNAs by sponging mmu-miR-326-5p, mmu-miR-761, mmu-miR-1907, or mmu-miR-3473. It was notable that one of the target mRNAs, Birc5, promotes VEGF expression, regulates the expression of angiogenesis-associated factors, and increases endothelial angiogenesis and migration (20). In addition, extensive studies have proposed Lgals3 as a regulator of cell proliferation, apoptosis, adhesion, and migration, which affects many aspects of biological processes such as angiogenesis and tumorigenesis (21). Besides, circRNA.7078 may regulate the expression levels of Top2a, Cd44, Ms4a6c, and KNSTRN by sponging mmu-miR-761, mmu-miR-1907 or mmu-miR-3473, and mmu-miR-5710. Top2a has previously been reported as one of the top 10 core genes in a SCI model at 2 days after spinal cord injury (22). Top2a is expressed in proliferating cells during the S and G2M phases of the cell cycle, and is primarily involved in regulation of cell proliferation (23). circRNA.6777 may regulate the expression levels of Fn1 by sponging mmu-miR-7684-5p. Extensive studies investigating Fn1 have shown that it inhibits apoptosis and regulates EMT to promote melanoma proliferation and metastasis (7). Furthermore, Fn1 also participates in colorectal carcinogenesis by promotion vascular endothelial proliferation, migration, and invasion (24).

Angiogenesis is central to a number of pathophysiological processes, from embryogenesis to wound healing (25). It is a complex process including extensive interaction between endothelial cells (EC), soluble factors, and extracellular matrix components. It has been reported that vascular-related events such as vascular endothelial proliferation, migration, and angiogenesis promote the recovery of SCI (26). Previous studies have suggested that CD31 (PECAM-1) is involved in angiogenesis and plays an important role in the formation of new vessels by interaction with other endothelial cell-cell adhesion molecules (27). As a main angiogenetic factor, vascular endothelial growth factor A (VEGFA) promotes endothelial cell proliferation after injury in various types of tissue, including the central nervous system (28). It has been reported that the VEGF signaling pathway, initiated by VEGFA/VEGFR2, leads to endothelial cell proliferation, migration, survival, and new vessel formation (29). It is worth noting that endothelial cells start newly forming microvessels as early as 3 days after injury, restoring the density of microvessels to normal levels by 1 week after SCI (30). In our present study, western blot analysis confirmed a decreased protein expression of CD31, VEGF, and VEGFR2 3 days after SCI. Therefore, we propose that microvessels are destroyed during the early 3 days after SCI, after which endogenous signaling pathways are gradually activated to re-initiate vascular endothelial proliferation, migration, and angiogenesis. Based on KEGG pathways analysis, we found that 10 endogenous signaling pathways were associated with vascular endothelial proliferation, migration, and angiogenesis. The most altered circRNAs were enriched for ECM-receptor interaction and the PI3K-Akt signaling pathway. We were particularly interested in the VEGF signaling pathway host genes of altered circRNAs. We found that eight host genes of circRNAs, including PLCy, PKC, SPK, Ras, NFAT, COX2, MAPKAFK, and HSP27 were involved in the VEGF signaling pathway.

In our previous study, we revealed the circRNAs related to the progress of apoptosis after SCI (15). Apoptosis and angiogenesis are two different but important biological events after SCI. Apoptosis is mainly related to injury. However, angiogenesis is mainly related to repair and regeneration. Recently, some circRNAs have been reported to play a role in vascular endothelial proliferation, migration, and angiogenesis. For example, circRNA hsa_circ_0003575 induces vascular endothelial cell proliferation and angiogenesis by regulating oxLDL (31). It has been reported that upregulation of circRNA-MYLK promotes the growth, angiogenesis, and metastasis of bladder carcinoma xenografts (32). A recent study indicated that downregulation of circNfix promotes cardiomyocyte proliferation and angiogenesis while inhibiting cardiomyocyte apoptosis after myocardial infarction, attenuating cardiac dysfunction and improving the prognosis (33). However, the circRNAs involved in vascular endothelial proliferation, migration, and angiogenesis after SCI have not yet been clarified. Thus, we paid special attention to the roles of circRNAs that were related to angiogenesis after SCI. In this study, a new batch of SCI mice was used for independent sequencing. This independent sequencing result was approximately consistent with our previous data, which proves the reliability of the sequencing results. On the basis of reliable sequencing results, we revealed the function of circRNAs in angiogenesis for the first time. We also correlated mRNA and circRNA expression using ceRNA prediction in order to identify circRNAs related to vascular endothelial proliferation, migration, and angiogenesis based on the GO and KEGG analysis. As a result, we found 93 circRNAs involved in vascular endothelial proliferation, migration, and angiogenesis. These findings may be helpful to more thoroughly understand the mechanisms of the early stages of SCI and might present novel molecular targets for clinical therapy of SCI in the future.

## Conclusion

Our data suggest that the circRNAs circRNA.7079, circRNA.7078, and circRNA.6777 may affect vascular endothelial proliferation, migration, and angiogenesis after SCI. Our findings may provide new potential strategies for the treatment of SCI via circRNA modulation.

## Data Availability Statement

The datasets presented in this study can be found in online repositories. The names of the repository/repositories and accession number(s) can be found below: National Center for Biotechnology Information (NCBI) Gene expression Omnibus (GEO), https://www.ncbi.nlm.nih.gov/geo/, GSE166967.

## Ethics Statement

The animal study was reviewed and approved by Zhejiang University School of Medicine.

## Author Contributions

FZ and L-lW conceived and designed this study. XY, JW, and YY performed the experiments. XY and YC wrote the manuscript. JC revised the manuscript. All authors contributed to the article and approved the submitted version.

## Conflict of Interest

The authors declare that the research was conducted in the absence of any commercial or financial relationships that could be construed as a potential conflict of interest.

## Publisher's Note

All claims expressed in this article are solely those of the authors and do not necessarily represent those of their affiliated organizations, or those of the publisher, the editors and the reviewers. Any product that may be evaluated in this article, or claim that may be made by its manufacturer, is not guaranteed or endorsed by the publisher.

## References

[1] Singh A Tetreault L Kalsi-Ryan S Nouri AF Ehlings MG. Global prevalence and incidence of traumatic spinal cord injury. Clin Epidemiol. (2014) 6:309–31. 10.2147/CLEP.S68889 25278785 PMC4179833

[2] Noonan VK Fallah N Park SE Dumont FS Leblond J Cobb J. Health care utilization in persons with traumatic spinal cord injury: the importance of multimorbidity and the impact on patient outcomes. Top Spinal Cord Injury Rehabil. (2014) 20:289–301. 10.1310/sci2004-289 25477742 PMC4252129

[3] Dvorak MF Noonan VK Fallah N Fisher CG Rivers CS Ahn H. Minimizing errors in acute traumatic spinal cord injury trials by acknowledging the heterogeneity of spinal cord anatomy and injury severity: an observational Canadian cohort analysis. J Neuro. (2014) 31:1540–7. 10.1089/neu.2013.3278 24811484 PMC4161054

[4] Guttman M Amit I Garber M French C Lin MF Feldser D. Chromatin signature reveals over a thousand highly conserved large non-coding RNAs in mammals. Nature. (2009) 458:223–7. 10.1038/nature07672 19182780 PMC2754849

[5] Jia H Ma H Li Z Chen F Fang B Cao X. Downregulation of LncRNA TUG1 inhibited TLR4 signaling pathway-mediated inflammatory damage after spinal cord ischemia reperfusion in rats via suppressing TRIL expression. J Neuropathol Exp Neurol. (2019) 78:268–82. 10.1093/jnen/nly126 30715406

[6] Zhou HJ Wang LQ Wang DB Yu JB Zhu Y Xu QS. Long noncoding RNA MALAT1 contributes to inflammatory response of microglia following spinal cord injury via the modulation of a miR-199b/IKKβ/NF-κB signaling pathway Am J Physiol Cell physiol. (2018) 315:C52–61. 10.1152/ajpcell.00278.2017 29631367

[7] Li B Shen W Peng H Li Y Chen F Zheng L. Fibronectin 1 promotes melanoma proliferation and metastasis by inhibiting apoptosis and regulating EMT. Onco Targ Ther. (2019) 12:3207–21. 10.2147/OTT.S195703 31118673 PMC6503329

[8] Xu L Ye X Zhong J Chen YY Wang LL. New insight of circular RNAs' roles in central nervous system post-traumatic injury. Front Neurosci. (2021) 15:644239. 10.3389/fnins.2021.644239 33841083 PMC8029650

[9] Jeck WR Sharpless NE. Detecting and characterizing circular RNAs. Nat Biotechnol. (2014) 32:453–61. 10.1038/nbt.2890 24811520 PMC4121655

[10] Chen LL Yang L. Regulation of circRNA biogenesis. RNA Biol. (2015) 12:381–8. 10.1080/15476286.2015.1020271 25746834 PMC4615371

[11] Memczak S Jens M Elefsinioti A Torti F Krueger J Rybak A. Circular RNAs are a large class of animal RNAs with regulatory potency. Nature. (2013) 495:333–8. 10.1038/nature11928 23446348

[12] Jeck WR Sorrentino JA Wang K Slevin MK Burd CE Liu J. Circular RNAs are abundant, conserved, and associated with ALU repeats. RNA. (2013) 19:141–57. 10.1261/rna.035667.112 23249747 PMC3543092

[13] Lukiw WJ. Circular RNA (circRNA) in Alzheimer's disease (AD). Front Genet. (2013) 4:307. 10.3389/fgene.2013.00307 24427167 PMC3875874

[14] Cortés-López M Miura P. Emerging functions of circular RNAs. Yale J Biol Med. (2016) 89:527–37. 28018143 PMC5168830

[15] Yao Y Wang J He T Li H Hu J Zheng M. Microarray assay of circular RNAs reveals cicRNA.7079 as a new anti-apoptotic molecule in spinal cord injury in mice. Brain Res Bull. (2020) 164:157–71. 10.1016/j.brainresbull.2020.08.004 32882320

[16] Zhang S Zhu D Li H Li H Feng C Zhang W. Characterization of circRNA-Associated-ceRNA networks in a senescence-accelerated mouse prone 8 brain. Mol Ther. (2017) 25:2053–61. 10.1016/j.ymthe.2017.06.009 28669840 PMC5589091

[17] Mehta SL Pandi GV Emuganti R. Circular RNA expression profiles alter significantly in mouse brain after transient focal Ischemia. Stroke. (2017) 48:2541–8. 10.1161/STROKEAHA.117.017469 28701578 PMC5575968

[18] Bai Y Zhang Y Han B Yang L Chen X Huang R. Circular RNA DLGAP4 ameliorates ischemic stroke outcomes by targeting miR-143 to regulate endothelial-mesenchymal transition associated with blood-brain barrier integrity. J Neurosci. (2018) 38:32–50. 10.1523/JNEUROSCI.1348-17.2017 29114076 PMC6705810

[19] Jiang YJ Cao SQ Gao LB Wang YY Zhou B Hu X. Circular ribonucleic acid expression profile in mouse cortex after traumatic brain injury. J Neurotr. (2019) 36:1018–28. 10.1089/neu.2018.5647 30261810

[20] Shang X Liu G Zhang Y Tang P Zhang H Jiang H. Downregulation of BIRC5 inhibits the migration and invasion of esophageal cancer cells by interacting with the PI3K/Akt signaling pathway. Oncol Lett. (2018) 16:3373–9. 10.3892/ol.2018.8986 30127937 PMC6096085

[21] Hayashi Y Jia W Kidoya H Muramatsu F Tsukada YT Akakura N. Galectin-3 inhibits cancer metastasis by negatively regulating integrin β3 expression. Am J Pathol. (2019) 189:900–10. 10.1016/j.ajpath.2018.12.005 30653955

[22] Shi Z Ning G Zhang B Yuan S Zhou H Pan B. Signatures of altered long noncoding RNAs and messenger RNAs expression in the early acute phase of spinal cord injury. J Cell Physiol. (2019) 234:8918–27. 10.1002/jcp.27560 30341912

[23] Kozuki T Chikamori K Surleac MD Micluta MA Petrescu AJ Norris EJ. Roles of the C-terminal domains of topoisomerase IIα and topoisomerase IIβ in regulation of the decatenation checkpoint. Nucleic Acids Res. (2017) 45:5995–6010. 10.1093/nar/gkx325 28472494 PMC5449615

[24] Zhang H Sun Z Li Y Fan D Jiang H. MicroRNA-200c binding to FN1 suppresses the proliferation, migration and invasion of gastric cancer cells. Biomed Pharmacother Biomed Pharmacother. (2017) 88:285–92. 10.1016/j.biopha.2017.01.023 28113080

[25] Zhou T Zheng Y Sun L Badea SR Jin Y Liu Y. Microvascular endothelial cells engulf myelin debris and promote macrophage recruitment and fibrosis after neural injury. Nat Neurosci. (2019) 22:421–35. 10.1038/s41593-018-0324-9 30664769 PMC6913093

[26] Mautes AE Weinzierl MR Donovan F Noble LJ. Vascular events after spinal cord injury: contribution to secondary pathogenesis. Phys Ther. (2000) 80:673–87. 10.1093/ptj/80.7.673 10869130

[27] DeLisser HM Christofidou-Solomidou M Strieter RM Burdick MD Robinson CS Wexler RS. Involvement of endothelial PECAM-1/CD31 in angiogenesis. Am J Pathol. (1997) 151:671–7. 9284815 PMC1857836

[28] Sköld M Cullheim S Hammarberg H Piehl F Suneson A Lake S. Induction of VEGF and VEGF receptors in the spinal cord after mechanical spinal injury and prostaglandin administration. Eur J Neurosci. (2000) 12:3675–86. 10.1046/j.1460-9568.2000.00263.x 11029637

[29] Abhinand CS Raju R Soumya SJ Arya PS Sudhakaran PR. VEGF-A/VEGFR2 signaling network in endothelial cells relevant to angiogenesis. J Cell Commun Sig. (2016) 10:347–54. 10.1007/s12079-016-0352-8 27619687 PMC5143324

[30] Chen MH Ren QX Yang WF Chen XL Lu C Sun J. Influences of HIF-lα on Bax/Bcl-2 and VEGF expressions in rats with spinal cord injury. Int J Clin Exp Pathol. (2013) 6:2312–22. 24228092 PMC3816799

[31] Li CY Ma L Yu B. Circular RNA hsa_circ_0003575 regulates oxLDL induced vascular endothelial cells proliferation and angiogenesis. Biomed Pharmacother Biomed Pharmacother. (2017) 95:1514–9. 10.1016/j.biopha.2017.09.064 28946214

[32] Zhong Z Huang M Lv M He Y Duan C Zhang L. Circular RNA MYLK as a competing endogenous RNA promotes bladder cancer progression through modulating VEGFA/VEGFR2 signaling pathway. Cancer Lett. (2017) 403:305–17. 10.1016/j.canlet.2017.06.027 28687357

[33] Huang S Li X Zheng H Si X Li B Wei G. Loss of super-enhancer-regulated CircRNA Nfix induces cardiac regeneration after myocardial infarction in adult mice. Circulation. (2019) 139:2857–76. 10.1161/CIRCULATIONAHA.118.038361 30947518 PMC6629176

